# Conspiracy Narratives as a Type of Social Myth

**DOI:** 10.1007/s10767-023-09454-1

**Published:** 2023-06-10

**Authors:** Radek Chlup

**Affiliations:** Department of Philosophy and Religious Studies, Faculty of Arts, Charles University, nam. J. Palacha 2, 116 38 Prague, Czech Republic

**Keywords:** Conspiracy theory, Social myths, Political myths, Fictional myths, Politics

## Abstract

It has long been recognized that conspiracy narratives may be seen as a special kind of myth. In most cases, however, this is taken as a sign of their irrational and unsubstantiated nature. I argue that mythical modes of reasoning are actually far more pervasive in modern political and cultural discourse than we commonly admit and that the difference between mainstream discourse and conspiracy narratives is not one between “rational” and “mythical” thought but rather one between different types of mythical thinking. The specific nature of conspiracy myths is best understood in relation to two other types of social myths: political myths and fictional myths. Conspiracy myths are a hybrid of these two genres: like fictional myths, they make use of imaginative elements, but like political myths, they are understood as having a relatively straightforward relation to reality and not just a metaphorical one. They are essentially anti-systemic, and their chief ethos is that of distrust. Nevertheless, the degree to which they reject the system varies, and it is thus useful to distinguish between weaker and stronger conspiracy myths. While the latter reject the system altogether and are incompatible with political myths, the former are capable of co-operating with them.

It has long been recognized that conspiracy theories can be fruitfully conceptualized as a special kind of *cultural narrative* (Butter, 2014; Fenster, 2008; Tangherlini et al., 2020; Bonetto and Arciszewski, 2021; Carver et al., 2022). “At the heart of conspiracy theory” there is “a gripping, dramatic story” accusing a group of powerful persons of secretly conspiring against the common good and doing so in a manner that frequently attempts “to explain a wide range of seemingly disparate past and present events and structures within a relatively coherent framework” (Fenster, 2008, p. 119). Conceptualizing conspiracy theories as narratives has at least two advantages. First, it allows us to see them as linked to various conspiracy plots in contemporary films, TV series, and novels and thus to recognize them as a wider cultural phenomenon that in recent decades has shifted “from the fringe to the mainstream of American political and cultural life” (Knight, 2000, p. 36). Second, in accordance with the narrative turn of the 1980s, which sees narratives as “an instrument of mind in the construction of reality” (Brunner, 1991, p. 6), we may now view conspiracy theories as collective meaning-making devices rather than just unsubstantiated flights of fancy. This allows us to compare them with other types of cultural narratives and to analyze their different sociopolitical outcomes, a project compatible with the strong program in cultural sociology (Alexander & Smith, 2010).

In my paper, I would like to build on this approach and develop it by conceptualizing conspiracy narratives as a specific type of *social myth*. That conspiracy narratives are in many respects heirs to religious myths has been noted by a number of scholars (Girardet, 1986; Madisson, 2014; Robertson and Dyrendal, 2019). In most cases, however, this is understood in the spirit of the “pathologizing paradigm“ that sees conspiracy myths not just as something imaginary and unsubstantiated (“the myth of a Jewish conspiracy“) but even more importantly as something opposed to the rational project of modernity: “the secularization of a religious superstition“ (Popper, 2013 [1945], p. 306), a revival of “archaic models of consciousness” which happens in times of social stress when “habitual patterns of behavior stop functioning efficiently“ (Madisson, 2014, p. 286). I am aware that the term “myth” inevitably evokes such connotations of irrationality and superstition, but I believe that it can actually be used in a neutral manner, in line with the narrative turn. Following Bouchard (2017) and several other scholars, I will argue that there are important cultural narratives that, in addition to the meaning-making ability shared by all narratives, display further special features that can actually best be described as “mythical,” such as the ability to arouse strong emotions and exert a fascination on account of expressing deep cultural concerns and desires^1^. I will try to show that such mythical modes of reasoning are far more pervasive in modern political and cultural discourse than we commonly admit and that the difference between mainstream discourse and conspiracy narratives is not one between “rational“ and “mythical“ thought but rather one between different types of mythical thinking. In this way, I hope to throw new light on the part conspiracy narratives play in the discursive field of our late modern times.

## Theoretical Background: Modern Social Myths

That our mainstream political discourse bears strong mythical features was occasionally argued by individual scholars decades ago (Edelman, 1967; Tudor, 1972; Bennett, 1975), but it was only in the 1990s that this topic came to be investigated in a more systematic manner. Thanks to the pioneering work of the historical sociologist Anthony D. Smith, many British scholars started to map the fundamental role of historical myths in the formation of modern nations (Hosking & Schöpflin, 1997; Smith, 1999). Other scholars attempted to synthesize previous insights concerning the general role of myth in political discourse (Flood, 1996; Bottici, 2007).

All of these approaches, along with many others, were put together by the Canadian sociologist Gérard Bouchard (2013, 2017) in his conception of social myths. For Bouchard, myths lie at the core of all collective social identities. They differ from other kinds of narrative in that they not only meaningfully organize our experience and memory but do so in a particularly forceful manner. They “possess an authority akin to sacredness,“ cross the boundary between reason and emotion, “permeate the minds of individuals, touch them deep inside, and motivate their choices, either by mobilizing them, by sending them forth in pursuit of bold plans, or on the contrary by inhibiting them; … their hold on consciousness is such that, having been deeply internalized, they are taken for granted and surrounded by an aura that enables them in large part to avoid being questioned.“ (Bouchard, 2017, p. 8–9). They mobilize members of social groups around their collective goals and visions, but they may equally well evoke feelings of hopelessness or hatred.

While drawing on Bouchard’s conception, for the purpose of my article I will modify it slightly to fit the study of conspiracy narratives. My main question will be what conspiracy myths are and how exactly they differ from mainstream social myths. A similar question has already been asked by Girardet (1986) and Giry (2015), but both scholars try to answer it mainly by specifying typical content features of conspiracy narratives (which they see as one type of political myth). Girardet, for instance, distinguishes four kinds of political myth in terms of their plots: the myth of the golden age, of unity, of the savior, and of conspiracy. However, while this may be a valid typology, it does not seem sufficient for grasping the difference between conspiracy myths and mainstream social myths. It is telling that public discourse conspiracy narratives are often openly denounced as “mythical“ (meaning “irrational and unsubstantiated”), whereas the mythical nature of the mainstream social narratives is usually not recognized at all. Clearly, therefore, the difference will be a matter of different mythical *genres* rather than just narrative *plots*.

More promising for me is Bouchard’s own distinction between social myths sensu stricto and other types of myths, such as religious myths, allegorical myths, or scientific myths. These too are seen by him as social in a broad sense, but they are distinguished from social myths sensu stricto in that the latter “are born and develop entirely in the immediate social arena“ and are actively used by “social actors who very openly promote and use them, in a spirit of symbolic engineering“ (Bouchard, 2017, p. 32). Although I agree with this, I find it more practical to designate these types of narrative as *political myths*, retaining the broader label of “social myth“ for all myths that somehow reflect the sociocultural system and its structures and tensions. A social myth in this sense is any collective narrative which arouses strong emotions and “exerts a fascination” (Dabezies, 1992) on account of relating to the basic values and principles of sociocultural reality, and expressing deep concerns, anxieties, and desires related to them. To adapt the well-known definition of religion by Geertz (1966, p. 23), we may say that social myths “establish powerful, pervasive, and long lasting moods and motivations in men,“ clothing them “in such an aura of factuality that the moods and motivations seem uniquely realistic.“ At the same time, myths are not to be seen as clearly bounded entities but rather as a mythical quality that can be attached to various narratives, conceptions, values, or ideals, thus imbuing them with fascination. In other words, rather than distinguishing between myths and non-myths, I find it more useful to ask which cultural formations are more mythical and which are less so, based on the degree of fascination they are capable of invoking.

I argue that the specific nature of *conspiracy myths* is best understood in relation to two other types of social myths: *political myths*, on the one hand, and *fictional myths* (called “allegorical“ by Bouchard) on the other. While the former is expressed in political speeches or in various public appeals made by artists and intellectuals, the latter may be found in novels, films, or computer games. Conspiracy myths are a special mythical genre that mixes the features of both political and fictional myths, and it is precisely their hybrid quality that makes them so disconcerting.

## Political Myths

Political myths display all the characteristics of social myths as defined above but are more specifically characterized by three features: (1) they function as a warrant or a “charter” (Malinowski, 1926) that directly legitimizes some of the values and principles of a given community. (2) In political myths, the fascination that social myths in general exert takes more specifically the form of *sacredness*: they evoke something highly authoritative and incontestable for the social group, using this for legitimizing political proposals. “Sacred rhetoric“ makes political convictions absolute and unassailable, invoking moral outrage at their perceived violations (Marietta, 2008). (3) They imply some kind of mobilizing moral message for both the present and the future. “Political myths are stories that make their moral explicit in order to prompt political action“ (Bottici, 2007, p. 215–216). A successful political myth has an “activation effect,” increasing political engagement (Marietta, 2008).

The pattern may best be seen in various historical myths which narrate historical events in a dramatic manner that reaches some kind of moral “closure“ with mobilizing potential. A classic example is the grand national myths created by nineteenth-century historians to legitimize the political identity of their respective nations, which indeed exerted a strong fascination on the populations in question and evoked feelings akin to sacredness (Smith, 1999). But the same pattern may be seen even in various recent “smaller“ historical myths, for example, those concerned with WWI–II or the Communist period in Central and Eastern Europe. Dramatic stories of these events are recounted again and again, in this way imparting tragic emotions even to younger generations who have not experienced these events themselves.

As Bouchard explains (2017, p. 49–53), while outstanding historical events serve as the myth’s “anchor,“ what really defines a good myth is the emotional “imprint“ that the narrative is able to produce in the listeners. A good myth is capable of reactivating these emotions at any time and turning them into what Bouchard (2017, p. 53) calls an “ethos,“ i.e., a moral message that is to be drawn from history and that relates to the present, “a set of aspirations, beliefs, principles, values, ideals, moral standards, visions of the world, and attitudes, or deep predispositions.“ In many cases, the myth manages to establish a kind of “mystic participation“ between the past and the present, creating the impression that a past event is still fully alive and unfinished, waiting for us to complete it in our actions here and now. In this way, myths can mobilize members of the nation around its collective goals and visions, although they can equally well evoke feelings of hopelessness, or even prompt “resentment, hatred, desire for vengeance, and violence“ (Bouchard 2017, p. 53).

Political myths need not just be based on history. They may also recount various “universal“ *values*: democracy, freedom, gender equality, human rights, etc. Sometimes these values are historicized, depicted as deeply rooted in the nation’s past experiences (Bouchard 2017, p. 60). But they may equally well be linked to the *future*, as in various myths of social and cultural progress. Or they may simply be seen as something timeless and eternally valid. In such cases, political myths may occasionally lean on *religion*, as in the pro-life myth, or even more importantly, on *science*, which in modern times functions as a crucial source of authority. Powerful recent examples of scientific myths are the global warming myth or various myths associated with the COVID-19 pandemic. By designating these as “myths,“ I do not imply that they would be factually untrue. Just as historical myths typically narrate events that did actually happen, scientific myths do usually respect the established facts. What makes them mythical is the way they narrate about them: they do so to arouse emotions and generate a mobilizing ethos. A typical mainstream COVID-19 myth is catastrophic, warning of collapsing hospitals and freezer trucks full of dead bodies but then turning this emotional horror into a more hopeful moral message, urging people to save lives by complying with pandemic measures. In myths of this kind, facts are easily mixed with terrifying predictions (in most countries, hospitals did not collapse, and no freezer trucks were necessary). At the same time, the ethos is clothed in such a sacred aura of factuality that it seems unassailable and “uniquely realistic.“

A fundamental feature of political myths is their relatively *straightforward relation to reality*. We understand them as an authoritative framework for social and political action. They are used to legitimize political proposals and invoked frequently in political speeches and campaigns. A successful political myth deals with “real“ problems and proposes “real“ solutions to them. As Atkinson and DeWitt (2019, p. 129) argue (following Kingdon, 1984), the fundamental problem of politics is that of “galvanizing collective action“ around certain problems and their suggested solutions. Political myths have precisely the task of enabling this, increasing the engagement and active participation of citizens in politics (Marietta, 2008).

This means that political myths always need to be constructive to some degree, to suggest solutions to problems, and to evoke *trust* in these solutions as well as in the politicians who tell the myth. In other words, political myths tend to be relatively *pro-systemic* in one way or another. There are of course political myths that strongly criticize the present political system and its values, e.g., the myths told by various present-day European nationalist parties such as the French National Rally, the German Alternative for Germany, or the Czech Freedom and Direct Democracy. Yet, while these stand outside the political mainstream, they cannot reject the system altogether and need to offer some positive vision of its reform (unrealistic as it may sometimes be), or else they would not be able to galvanize collective action. Occasionally, there may even emerge radically anti-systemic myths, such as the “revolutionary myth“ that Sorel chose as the prime example of a political myth. Still, even Sorel saw revolutionary violence as only a temporary stage “at the service of the immemorial interests of civilization“ which “may save the world from barbarism“ (Sorel, 1999 [1908], p. 85), i.e., even his myth was driven by a positive vision to be accomplished, with anti-systemic destruction being just a way leading to it.

The imperative of galvanizing collective action means that political myths also tend to be relatively *established and accepted*, though perhaps just by a group of voters. And if they are not widely accepted, they need to have this at least as their aim. Even Sorel’s revolutionary myth has as its chief task to mobilize the workers, crystallize their latent aspirations, and turn them to action. The more successful a political myth is, the more people it can incite to action. In this regard, extremist myths with few adherents, while no doubt capable of arousing intense emotions, represent a marginal and unsuccessful form of political myth which fails to fulfill some of their basic functions.

## Fictional Myths

Whereas “political myths“ are a relatively established concept, “fictional myths“ are much less so. Most modern accounts of the relationship between myths and literature deal with the origin of the latter in the former. Literature has sprung out of myth, and in its basic images and plots, it still bears great resemblance to it. As Frye classically put it (1957, p. 134–135), the “structural principles of literature are as closely related to mythology and comparative religion as those of painting are to geometry.“ While in modern times many types of fiction have moved far away from the kind of fantastic stories and images that we find in traditional myths, in recent decades, there has been a revival of explicit mythical references in the fantasy genre. Works such as *Star Wars* or *The Lord of the Rings*, as well as numerous computer games, are directly inspired by ancient mythologies.

However, to function as a myth, a fictional narrative need not contain traditional mythical motifs at all. From my perspective, what matters is that it is capable of capturing the minds and souls of the audience, of expressing their deep concerns, anxieties, and desires, of formulating an ethos, and of doing this in a manner that makes all of this seem “uniquely realistic.“ My conception of a fictional myth thus comes close to how Dabezies (1992, p. 961–962) defines literary myth:

a symbolic narrative ... which exerts a fascination (whether idealistic or repulsive), as well as a greater or lesser power to bring people in a large or small human community together by offering them the explanation for a situation or a call to action. ... A literary text is not in itself a myth: it returns to and re-expresses mythical images and can itself acquire mythical value and fascination in such circumstances, for a particular audience at a particular time. In the same way it can lose this mythical value when the audience or circumstances change. ... Thus a simple literary “theme“ acquires the status of a myth when it expresses the mental constellation in which a social group recognizes itself (for example, the figure of Tristan in the twelfth century, for a small fringe of the courtly aristocracy), and when it no longer fascinates the public it once more becomes a simple theme, to which writers return merely out of habit or literary tradition (Tristan in the fifteenth or sixteenth centuries).

At first sight, fictional myths may appear as completely unconnected to political myths. While the latter relate to the “real“ world, the former belong to the realm of fiction and imagination. Indeed, in our times, some of the most influential fictional myths are those that are from the outset situated in fantasy lands completely different from our ordinary world. Nevertheless, there are certain features that political and fictional myths have in common. Like political myths, fictional myths are relatively *established and accepted*, despite the fact that they occasionally offer radical criticism of our mainstream reality. *The Matrix* is a case in point: taken at its face value, the film presents our everyday world as a technologically generated illusion that allows the actual imprisonment and abuse of humans and their bodies. If such a claim were made by someone outside the film screen, they would immediately be labeled as “conspiracy theorists.“ Yet, once the same story is situated in the realm of fiction, it becomes perfectly acceptable.

In this regard, fictional myths are also similar to political myths in being more or less *pro-systemic* and *accepted*, for otherwise they would not resonate with mainstream audiences and would lose cultural significance. There are, of course, significant differences: a romantic comedy will be basically in harmony with the dominant sociocultural order, while a post-apocalyptic movie will voice strong criticism of it. However, while normally such criticism might be seen as dangerous, the label of “fiction“ makes it safe, preventing it, as it were, from uncontrollably spilling over into reality. In effect, the anti-systemic dimension of novels and films is mild and harmless. They resemble jokes, which have “subversive effect on the dominant structure of ideas“ (Douglas, 1975, p. 95), but the subversion only lasts for a moment: once the laughter cools down, we may safely return to the conventional structures of our social world.

For the same reason, we grant fictional myths the right to make things up in a fantastic manner without denouncing them as “disinformation.“ In fact, it is chiefly in the safe realm of fiction that we are still able to experience various supernatural powers that have long been banished from the everyday disenchanted world of modernity. As Roeland et al. put it (2012, p. 419), “fictional narratives ... are much more ‘disenchantment-proof’ than doctrinal and theistic Christianity, which after all requires ‘belief’ (in the sense of ‘placement beyond doubt and scrutiny’) and hence conformity to religious doctrines and authorities. Precisely because of their explicitly fictional status, the popular fictions of fantasy culture ... do not demand belief and conformity to doctrine.“

Nevertheless, while safely detached from our everyday modern reality, fictional myths do have something intensely real about them. They are capable of touching us deeply. The artists capture the typical feelings, aspirations, and anxieties of their time. Even commercial novels, films, and computer games draw their popularity from their articulation of something that deeply concerns their audience. Moreover, they have a strong performative force as well: they do not just *describe* reality, they also help to *create* it. They influence our thoughts, perceptions, and emotions. They are capable of generating precisely the kind of moral ethos that we have seen as one of the cornerstones of social myths. In this way, even fantasy stories seemingly unrelated to our present-day world may in fact relate to it indirectly. They “may inspire a spiritual search for meaning against the background of cultural discontents“ (Roeland et al., 2012, p. 419). And they may even “provide a sense of ‘home’ and feeling of belonging because they provide the opportunity to build small tribal communities,“ e.g., in LARPs or online game groups (Roeland et al., 2012, p. 411).

Still, the *relation to reality* that fictional myths exhibit differs sharply from that of political myths in being *indirect* only. It is for this reason that Bouchard (2017, p. 28–29) designates fictional myths as “allegorical,“ i.e., as relating to reality in an indirect manner through symbolic fictional figures and actions. This limits the political use of fictional myths. While a historical myth may be invoked as an authoritative argument in a parliamentary speech, one might hardly legitimize a political proposal with a pathetic appeal to *Harry Potter* or *Star Wars*. All we could do in such a situation is to use fictional images as rhetorical figures and *metaphors*, labeling a dangerous politician “Lord Voldemort,“ denouncing a naive political project as “Quixotic,“ speaking of corruption as succumbing to “the dark side of the Force,“ or referring to Russian troops invading Ukraine as “orcs.“ In contrast to this, political myths are used *metonymically*: a reference to the glorious deeds of the US “Founding Fathers“ or the terrible “Trianon Trauma“ of Hungary in a political speech is usually not just meant as an analogy but as expressing a more direct association or contiguity, as capturing an ethos that is still very much alive in contemporary politics.

What exactly does the relation of fictional myths to reality consist in? A possible (though by no means exhaustive) answer is offered by Boltanski (2014) in his analysis of detective novels and spy novels. In Boltanski’s view, these novels describe not the factual world of “everything that happens“ but rather *reality* in the sense of an ideal system of regularities that is presumed to be relatively coherent, stable, and foreseeable (Boltanski, 2014, p. 3, 10). Fictional myths may help to articulate such ideal conceptions of reality, but they usually also reflect their limits and help us to deal with the tensions and anxieties these limits produce in us. Every conception of reality is, of necessity, limited, entailing various internal contradictions. In traditional societies, as Lévi-Strauss has shown (1955, p. 443), these were frequently thematized in myths, which circled around the various cultural contradictions. By arranging the contradictions in symmetrical patterns, they made them cognitively manageable, though usually without really resolving them. Modern novels and films often do the same. Since the modern conception of reality, closely tied with science and the bureaucratic nation-state, is far more regular and coherent, our fictional narratives frequently do manage to resolve the contradictions to some extent, reaching some sort of a temporary happy ending.

In the case of detective and spy novels, one of these contradictions involves the question of truth (Boltanski, 2014, p. 20–21). In the large and complex modern nation-state, there is a huge distance between state officials and ordinary people, and it is thus difficult to tell when we are to believe what the authorities say, or whether they are hiding some other secret interest. This tension is explicitly thematized in spy fiction, but we may also find it in detective novels, in which everyone is a potential suspect and we are never certain who is telling the truth. Detective novels dramatize this anxiety, making it visible by means of a mysterious crime, which disturbs the regular outer face of reality and shows it as fragile despite its apparent robustness. The uncertainty surrounding the circumstances of the crime casts doubt on the state’s ability to maintain the regular order of reality. The task of the detective is to restore this order by demonstrating the crime as fully causally deducible from various visible signs and clues. In this way, detective novels “harness the anxieties, the tensions and even the contradictions that inhabit the relation between the political order and reality“ (Boltanski, 2014, p. 19–20).

Detective novels relate to reality in a highly specific manner, but it is not difficult to see each genre of fictional myths as related to reality in one way or another—sometimes by mapping its structures and the tensions connected with them, at other times by expressing alternatives that have been repressed from the official conception of reality (in this way resembling the part played by dreams in psychoanalysis).

## Conspiracy Myths

Whereas fictional narratives only turn into myths relatively rarely (most novels and films do not attain the collective fascination status), conspiracy narratives display mythical characteristics much more often. Their basic plot of uncovering a secret conspiracy that allows us to give deeper unifying meaning to a wide range of seemingly unconnected events does indeed make these narratives fascinating. Moreover, as Fenster shows, conspiracy narratives display a mesmerizing dynamics that arises out of a tension between the search for a grand final explanation and the impossibility of reaching it. Whenever a possible conspiracy is discovered, it faces a number of problems that require further search. “Conspiracy theory demands continual interpretation. There is always something more to know about an alleged conspiracy” (Fenster, 2008, p. 94). In the end, the search turns out to be endless. The conspiracy thus appears as “an enormous structure always on the horizon of interpretation, always the cause of everything, always the point toward which interpretation moves but which it never fully reaches” (Fenster 2008, p. 103–104). In this regard, conspiracy narratives always point beyond themselves, implying a kind of transcendence. It is not surprising, therefore, that they so easily combine with spirituality and acquire regular religious characteristics (Robertson & Dyrendal, 2019). All of this makes them ideal candidates for functioning as social myths.

As a mythical genre, conspiracy myths are to some extent similar to fictional myths, as they are not afraid of imaginative elements that are difficult to substantiate by facts. Yet the conspiracists themselves treat their narratives as if they were a type of political myths, i.e., they attribute to them a relatively straightforward relation to reality. We may thus say that conspiracy myths are *fictional myths taken metonymically* and not just metaphorically. Thus, for example, the conspiracist Icke (2001) no longer reads *The Matrix* as just an image expressing the frustrations of late modern social life by way of analogy but understands it as describing a deeply real “vibrational prison“ controlling all of our thoughts and perceptions and enslaving us in a system of illusions. “The truth is, the Matrix was a documentary,“ runs a quote frequently attributed to Keanu Reeves on conspiracist websites.

This is not to say that we should take such claims literally. From my mythological perspective, the relation of conspiracy myths to reality is closer to that we have identified for fictional myths: at heart, they are a symbolic reflection of the limits of our system and the tensions it contains. Yet, whereas fictional myths reflect on the limits of reality from a distance, so to speak, so that in many cases we are just enjoying the stories and are not even aware of any reflection going on, conspiracy myths make their critical relationship to reality more obvious, and their effect on the audience is thus much more disturbing.

Conspiracy myths are also a crossover between political and fictional myths in terms of their content. Like political myths, they are basically about history, universal values, and especially science. They are rationalistic and embrace critical thought and skepticism, and they are careful to emulate the scientific genre by making use of its terminology and providing footnotes for their sources. At the same time, however, their scientific discourse is much closer to science fiction than to science as such, and in its most developed forms, they resemble the baroque worlds of fantasy novels and films. In this way “they defy the typical distinction between skepticism and belief; the secular and the sacred; disenchantment and re-enchantment on which a modern culture is based, ... and combine the best of both worlds“ (Aupers, 2012, p. 31).

In this regard, conspiracy myths represent the ineradicable “grey zones“ of modernity (Ivakhiv, 2018). As Latour (1991) has famously argued, modernity has been based on a clear separation of different spheres of life: nature has been separated from culture, religion from science and politics, facts from values, etc. In fact, however, such clear-cut divisions have never worked, for there have always been various hybrids crossing the boundaries. Conspiracy myths may be seen as precisely one of these hybrids, mixing fact with fiction and metaphor with metonymy. Although even political myths are hybrid to some extent, mixing facts with values, emotions, and imaginative elements, the hybridity of conspiracy myths is much more fundamental, and it is for this reason that they are so disconcerting for most of us.

Where conspiracy myths differ from the other two types is in their distinctly *anti-systemic and anti-establishment* nature. In their purest form, they do not just see the established social and political system as impaired and in need of reform. They see it as essentially perverted, perceiving it as resulting from a malevolent conspiracy of dark powers. In this regard, conspiracism is an heir of the ancient Gnostics, who insisted that the divinity creating and ruling this world is not really the true God but an evil Demiurge enslaving us all with the help of his Archons. To see conspiracy myths as essentially anti-systemic might seem far from obvious, for there are of course numerous cases in which conspiracy myths do have a positive core, defending one type of sociocultural order against external agents threatening to disrupt it (McCarthyism being a classic example). I do not deny this, but as I explain below, from my perspective, such “scapegoating“ narratives represent a weaker form of conspiracism—“weaker“ not in the sense of being less intense but in that of mixing mythical genres and standing halfway between political myths and conspiracy myths. A strong conspiracy myth blames *the* system; a weaker one blames *a* system.

Hand in hand with the anti-systemic nature of conspiracy myths goes the fact that the narratives themselves are viewed by the mainstream as *illegitimate and rejected knowledge*. This is not to deny that there are numerous cases in which such narratives have been espoused by the state elites (again, the example of McCarthyism comes to mind). But even here this is best taken as an indication that such narratives are conspiracist only in a weak sense and are closer to political myths rather than to conspiracy myths in the strong sense of the term. It is significant that—as we shall see below—many of these narratives were not perceived as “conspiracy theories“ by their contemporaries, and it is only in retrospect that they are labeled as such.

An important feature of conspiracy myths is their ethos, which is generally that of *distrust* (Moore, 2019). Conspiracy myths are based on the expectation “that some other agent intends harm to you or your interests“ (Moore, 2019, p. 113). Such myths not only express distrust but are capable of generating it. We will see below what this means for their relation to politics.

## Mapping the Three Types of Myths

**Fig. 1:**
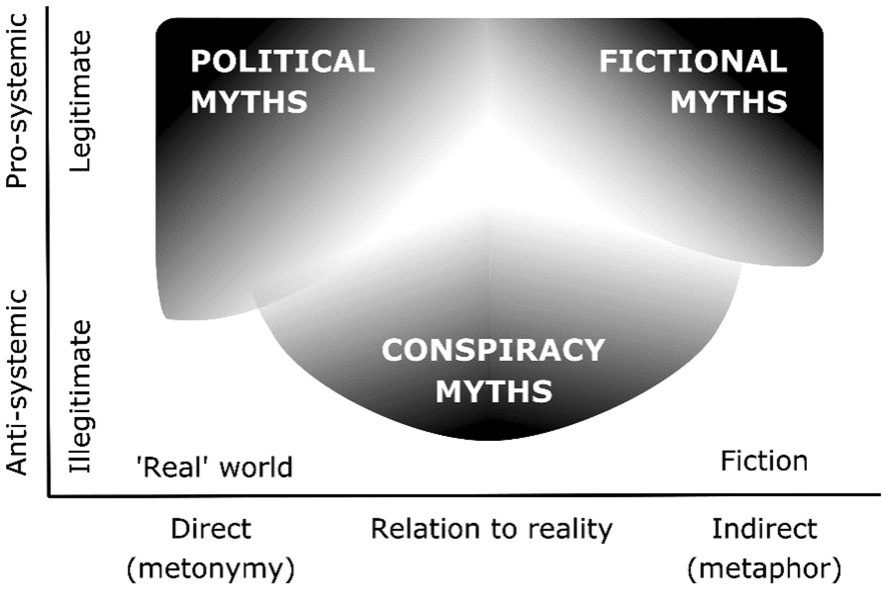
Mapping the three types of myth

The three types of social myths that I have presented should not be seen as clear-edged categories sharply separated from each other but rather as ideal extremes on a continuous scale of positions. We may perhaps map them in the following diagram (Fig. 1).

Each type of myth is depicted as an area stretching across the diagram and showing different degrees of purity (the darker the color, the purer the mythical genre in question). Political and fictional myths have their center of gravity at the upper pro-systemic end of the diagram, though both also appear in more anti-systemic versions (political myths more so than fictional myths, for reasons explained above). Conspiracy myths are radically anti-systemic at their purest, but they also have milder versions (lighter in color): in the upper right direction, the mildness consists in that some conspiracy narratives are less literal and more metaphorical than others (see below), and in the upper left direction, it lies in that some only distrust one kind of system, but not the system as such^2^.

Significantly, there are no clear boundaries between the three genres, and they all tend to overlap. Along the upper edge of the diagram, we find films and novels taking up various political myths and dramatizing them. Indeed, a political myth can probably never be successful without being adopted by the artists who popularize it. This may clearly be seen in various historical novels, poems, and films that from the nineteenth century onwards developed the new national myths (Walter Scott in Scotland, Adam Mickiewicz in Poland, Alois Jirásek in Czechia, etc.). Not being restrained by the requirements of facticity, historical novels, poems, films, and dramas are much more suitable for conveying the emotions and the moral ethos that form the basis of each political myth. A novelist can forcefully dramatize these features in the lives of individual characters by mixing historical facts with artistic creative license.

Of more interest to us is the right side of the diagram, marking the range between fictional and conspiracy myths. Here again the boundaries are far from clear. As cultural studies scholars have shown, conspiracy narratives are closely related to literary novels, films, and television shows (Knight, 2000; Melley, 2000). The *X-Files* TV series or the long tradition of conspiracy movies, from *The Parallax View* (1975) to *The Matrix* (1999) and *The Da Vinci Code* (2003), are the best-known examples. Many conspiracy motifs which would otherwise probably remain marginal were first popularized precisely by works of fiction. Thus, e.g., the reptilian narrative, which only became widespread in conspiracist circles in the 1990s, was first popularized by works such as the 1979 *Enemy Mine* novella (followed by a 1985 film adaptation) and the 1983 *V* television miniseries (Lewis and Kahn, 2005, p. 47).

Fictional conspiracy narratives have the advantage of being widely accepted, for they are perceived as not having a direct relation to reality but rather as being merely metaphorical. Yet, as Knight has shown (2000, p. 48), even in nonfictional conspiracy narratives, it is frequently difficult to decide to what extent they are taken literally. Conspiracy culture oscillates between the hoax and the accurate revelation, between the serious and the ironic, between the factual and the fictional, and between the literal and the metaphorical. In many instances consumers of conspiracy don’t really believe what they buy, but neither do they really disbelieve it either. Often people believe rumors with a provisional commitment, believing them *as if* they were true.

No less important is the blurring of boundaries on the left side of the diagram. What we find here is a continuous scale of positions that increasingly grow more and more critical of the entire social and political system. At first, the narratives focus on questioning some of the basic principles of the system without really offering any full-blown conspiracy myths. In the case of the COVID-19 pandemic, for instance, we would find here numerous attempts at undermining the faith in vaccination and other anti-pandemic measures. It is only gradually that incoherent hints at actual conspiracies start to emerge, such as those blaming large pharmaceutical companies (and Bill Gates in particular) for deliberately producing the pandemic in order to increase their profits. As we progress down the scale, the narratives begin to be increasingly developed and connected to a global conspiracy of the world’s elites who want to kill the freedom-loving part of the population and enslave the rest.

Even explicit conspiracy narratives may be arranged along a scale. As many scholars have noted (Knight, 2000; Melley, 2000; Aupers, 2012; Harambam, 2020), it is possible to distinguish two different types of conspiracy narratives. Before the 1960s, conspiracy narratives were generally based on scapegoating an exotic Other (e.g., Jews or communists), which led to “a paradoxically secure form of paranoia that bolstered one’s sense of identity“ (Knight, 2000, p. 4). As a result, these narratives were frequently told by mainstream politicians (Butter, 2020) and were thus very close to political myths. In the 1960s, alongside these classic modern narratives, there emerged a new type of conspiracy discourse which is no longer about defending our good community against the evil Other but rather about doubting the basic institutions of modern society itself. These “postmodern“ conspiracy narratives are characterized by “a far more insecure version of conspiracy-infused anxiety“ which stirs up “a permanent uncertainty about fundamental issues of causality, agency, responsibility and identity“ (Knight, 2000, p. 4). In this regard, they are much more anti-systemic than the classic scapegoating narratives, for they distrust the late modern sociocultural system as such. We may thus situate them at the lowest tip of our mythological map. Today, many conspiracy myths combine both classic modern and postmodern features: they picture an all-pervading conspiracy of the global elites, but at the same time, they still envisage some hope of fighting back and purging the nation of its enemies once again (e.g., in the “Storm“ operation predicted by the QAnon myth). Myths of this type would thus lie somewhere between McCarthy-like myths and postmodern conspiracy myths pure and simple.

## Implications for Defining Conspiracy Theory

The mythological approach just sketched has some interesting implications for the problem of how to define conspiracy theory. Scholars of conspiracism usually oscillate between two different strategies. The most common one is to define conspiracy theories in a *substantivist* manner by specifying their typical content features. Thus, for Barkun (2003, p. 3), a conspiracy belief is “the belief that an organization made up of individuals or groups was or is acting covertly to achieve some malevolent end,“ while for Uscinski (2019, p. 48) conspiracy theory “refers to an explanation of past, ongoing, or future events or circumstances that cites as a main causal factor a small group of powerful persons, the conspirators, acting in secret for their own benefit and against the common good.“ However, as Butter and Knight show (2020, p. 3–4), definitions of this kind involve several problems.

For one thing, does the “conspiracy theory“ label apply even to cases where the conspiracies have turned out to have actually happened, such as the Watergate affair? Clearly, this is not how we usually employ the term: in common usage, the “conspiracy theory“ label is only used when most people do not actually believe there is a conspiracy going on. A good example is the “COVID-19 lab leak“ theory which was originally labeled by many journalists and scientists as a “conspiracy theory“ (i.e., as a dangerous fanciful claim) but which subsequently started to be examined as a legitimate scientific hypothesis, which meant that the “conspiracy“ label was mostly dropped (Thacker, 2021). General disbelief in the validity of the theory thus seems to be one of the defining features of conspiracy theories. This means, however, that a conspiracy theory cannot be defined just by its own inherent properties but also by its place in the general discursive field.

Uscinski (2019, p. 48) tries to solve the problem by distinguishing between *conspiracy* as such, which “refers to events that our appropriate institutions have determined to be true,“ and *conspiracy theory*, which “refers to an accusatory perception which may or may not be true, and usually conflicts with the appropriate authorities.“ This certainly has the advantage of presenting a neutral and open-minded outlook on conspiracy theories, but it does not take sufficient account of the fact that “the term ‘conspiracy theory’ is anything but neutral in everyday discourse. Often, the label is used in a pejorative sense; it seems to provide a diagnosis of a flawed and delegitimized way of thinking, the aim of which is often simply to end discussion.“ (Butter & Knight, 2020, p. 3). While we, as scholars, should define concepts as neutrally as possible, the pejorative connotations are so widespread in public discourse that it might be better to make allowance for them in our definition.

Some scholars try to solve these problems by turning to a *functionalist* definition and delimiting conspiracy theories precisely by their position in the cultural field. This is the course most famously taken by Bratich (2008, p. 3), who insists that “[c]on﻿-spiracy theories are defined not merely by their strictly denotative, inherent properties, but by their discursive position in relation to a ‘regime of truth.’” The label of “conspiracy theories“ is used as “a sign of narrative disqualification“ (Bratich, 2008, p. 4) which tells us “less about the people who believe in them“ than it does “about the dominant forms of rationality that are so enraptured with them as problems“ (Bratich, 2008, p. 19). For Bratich, conspiracy theories are “dangerous knowledges“ that threaten the dominant regime of truth. In my terminology, they are narratives perceived as *anti-systemic*. Bratich certainly has a point, and his approach allows us to see something fundamental. It has been put to fruitful use, e.g., by Harambam (2020), who successfully identified the Dutch conspiracy milieu by first mapping what websites are labeled as “conspiracist“ by the mainstream media and then inquiring what other websites and theorists these “conspiracist“ sites and the people behind them considered their most valuable sources of news and information. In this way, he did indeed discover a relatively clearly delimited cultural anti-systemic milieu, though one that is scalar, and with blurred edges.

However, inspiring as such a position is, it has rightly been seen by other scholars as too extreme, suggesting “that conspiracy theories do not even really exist until the concept used to label them as such has emerged“ (McKenzie-McHarg, 2020, p. 17). Moreover, the functionalist approach seems impractical for mapping the gradual scale of the anti-systemic milieu and clearly distinguishing its various currents. In the USA, for instance, the Fox News channel spreads numerous anti-systemic political myths that doubt various present-day global mainstream narratives, such as those relating to climate change or anti-pandemic measures (Hoewe et al., 2020). Yet they rarely feature what we might be tempted to label as “conspiracy narratives“ (and if they do, it is mainly the milder scapegoating ones aimed against the Democrats—Perry, 2017). This is significantly different from full-blown conspiracy websites such as Alex Jones’ InfoWars, which regularly publish radical “postmodern“ conspiracy narratives on the plans of global elites to reduce the world population and throw the rest in slavery. To distinguish between these two anti-systemic narratives, it is quite handy to include in our definition the substantive element of a secret conspiracy of the powerful against the common good.

Therefore, I suggest that the substantivist and functionalist approaches should best be combined. It is useful to start with the substantivist approach and look for narratives that accuse a group of powerful persons of secretly conspiring against the common good. In the next step, however, we need to apply functionalist criteria as well. These are of two different kinds. (1) The first criterion follows Bratich in measuring the narrative’s position in the discursive field. We must ask by what sections of society it is accepted as possibly true and whether these sections represent the mainstream (the dominant “regime of truth“) or whether their views are generally viewed as marginal and anti-systemic. The more accepted the narrative is, the more it functions as a political myth instead of a conspiracy one. Thus, e.g., a conspiracy narrative accepted by a significant number of US Democrats or Republicans will be less conspiracist than the one told only by various alt-right groups. (2) The second criterion goes beyond Bratich, evaluating the way in which the narrative itself relates to the system. Does it see the entire sociopolitical system as corrupt beyond repair? Or does it see it as basically good but endangered by some dark forces that try to pervert it? In my model, the latter position will come out as less conspiracist than the former.

An interesting corollary of this approach is that it will probably detect a few strong conspiracy myths before the 1960s. This seems to be the case at least in the USA, where according to Butter (2020, p. 648–649) until the 1950s conspiracy theories were “officially accepted and legitimate knowledge“ which was “believed and articulated by elites,“ exerting “a significant influence on culture and society.“ It was only in the late 1950s that their status changed, and they began to be seen as “stigmatised and illegitimate knowledge.“ Thus, e.g., the “Red Scare“ was in the early 1950s “shared and seldom questioned by most liberals as well as conservatives“ (Fried, 1990, p. 36), which means that it was closer to a political myth than a conspiracy one. It was only at the end of the decade that the anti-communist suspicions ceased to be voiced by mainstream media and official government publications and came to be preached only by the marginal John Birch Society, in effect shifting toward the conspiracist end of the scale and at the same time assuming more and more fantastic and anti-systemic forms (Butter, 2020, p. 653–654). This goes hand in hand with the fact that it was only in the course of the 1950s that the concept of “conspiracy theory“ in today’s pejorative sense was established (Thalmann, 2019). While this does not mean that there were no conspiracy myths before the 1950s, they were much more integrated with mainstream political discourse, and thus, on my mythological map, they would be situated somewhere between political and conspiracy myths.

At the same time, scapegoating exotic Others as such should not be classed as conspiracism without the explicit substantive element of an alleged conspiracy plotted by these Others. Thus, an anti-immigration narrative only turns from a political myth into a conspiracy one when the immigrants are no longer just seen as dangerous on account of cultural incompatibility and criminal tendencies but when they start to be pictured as part of some kind of a malevolent plot of the elites to destroy our civilization (e.g., the “Great Replacement” of the freedom-loving Euro-American population with docile immigrants who are easier to enslave). The narrative motif of a conspiracy has the effect of deepening the ethos of distrust, for suddenly the danger embodied by the Others appears no longer as a random intrusion but more like a result of intentional systematic effort.

To sum up, conspiracy theories are narratives which accuse a group of powerful persons of secretly conspiring against the common good and which at the same time are anti-systemic with respect to both of their content and of their position in the mainstream discursive field. Importantly, the anti-systemicity is a matter of degree: it allows us to distinguish stronger conspiracy narratives from weaker ones and even to claim that some stories of secret conspiracies should not be classified as conspiracy theories at all.

## Conspiracy Myths and Politics

Since I claim that conspiracy myths are different in principle from political myths and yet they sometimes mix, it might be useful to examine how these two types of myths relate to each other and to what extent conspiracy myths may be used for political purposes. As a starting point, we may use the analysis of Enders and Smallpage (2019), who draw a useful distinction between two different types of psychological processes that people simultaneously have in relation to politics: *partisan or ideological reasoning* on the one hand (e.g., liberal or conservative views) and *conspiracy thinking* or *political suspicion* on the other (cf. Uscinski, 2020, p. 97–98). From my perspective, “ideological reasoning“ is precisely the province of political myths. “Conspiracy thinking,“ on the other hand, is a different type of discourse. Its basic principle is “a heated suspicion toward authority and a desire to distance oneself from the potentially oppressive powers of that authority“ (Enders & Smallpage, 2019, p. 312)—in other words, a distrust of the system.

This distrust or “political suspicion“ is a matter of degree. To some extent, most of us are slightly suspicious of the system, but the suspicion is too weak to translate into conspiracy narratives. Once it becomes stronger, conspiracy narratives offer themselves as its natural expression but at first only in the weaker form of the “scapegoating“ conspiracy myths. In this weaker form, conspiracism is actually quite common in US politics across the political spectrum (Enders & Smallpage, 2019; Uscinski & Parent, 2014). Both Republicans and Democrats have conspiracy theories of their own, accusing members of the other side of conspiring. As Uscinski and Parent argue (2014, p. 131), “conspiracy theories are essentially alarm systems and coping mechanisms to help deal with threats. Consequently, they tend to resonate when groups are suffering from loss, weakness, or disunity.“ In effect, conspiracy narratives get stronger whenever a party loses an election. Thus, when Trump won the election in 2016, more than 30% of Democrats started to believe that he conspired with Russia to influence the results, in this way articulating their feelings of powerlessness (Enders & Smallpage, 2019; Uscinski, 2020, p. 113–120). As Uscinski and Parent (2014) famously put it, “conspiracy theories are for losers.“

When political suspicion grows much stronger, it ceases to be compatible with political myths. At this point, the distrust no longer concerns just our political opponents but the entire system as such. This implies the rejection of standard political myths and situating oneself outside the mainstream. This is what we find in various extremist political groups, which have been shown to be much more prone to conspiracy thinking (van Prooijen et al., 2015).

At its strongest, political suspicion prevents any political involvement at all. This is an attitude that we may frequently encounter in various “conspiritual“ circles, which view politics as dirty in principle. As the Czech conspirituality Facebook page “Expanded Consciousness“ put it before the elections in October 2021 (https://www.facebook.com/rozsirenevedomi1111/posts/386807769782702): “The system is beyond reform. Whoever takes part in the elections will signal to the system that one still needs it for some reason. In this way, one will help the system survive.“

What this means is that political myths and conspiracy myths are uneasy bedfellows. They may sometimes cooperate, but there will always be a tension between them. Since the task of political myths is to bolster faith in political action, they require sufficient trust in the system and its reform. Conspiracy myths, on the other hand, are “both premised on and generative of distrust“ (Moore, 2019, p. 116). And while distrust may sometimes lead to active “opposition and popular vigilance,“ more often it leads to “defeatism and resentment,“ breeding “a negative dynamic, in which distrust promotes further distrust“ (Moore, 2019, p. 115–116). It is due to this “spiral of distrust“ that people who believe in one conspiracy theory are more likely to believe in others as well (Goertzel, 1994). For this reason, conspiracy myths are rather dangerous allies for politicians, for they may easily get out of control, undermining all political projects and aspirations.

It is true that, when used carefully, the politics of distrust may occasionally be turned by skillful politicians into a powerful weapon. As Atkinson and DeWitt argue (2019), due to their anti-systemic qualities, conspiracy theories can function as a game changer, a “disruptive innovation“ that reframes existing problems and changes the way people in the political system interact with one another. A case in point is the 2016 presidential campaign of Donald Trump, who as an outsider would normally have no chance of succeeding. Yet, by adopting a populist discourse that included various conspiracy narratives, he bypassed the conventional system and changed the rules of the game (Uscinski, 2020, p. 111–112). Nevertheless, as Atkinson and DeWitt admit (2019, p. 131), such “conspiracy theory politics“ is always dangerous for those who practice it, in as much as “the politics of disruption is different from the art of governance.“ In other words, the distrustful ethos bred by conspiracy myths may be useful for challenging the system, but in itself, it is not capable of reforming it. To do this, it needs to join hands with more conventional political myths. This is exactly what Trump did: “He may have successfully labeled himself as a populist disrupter, but his approach has hued more to the conservative narrative of the last 50 years than anything else“ (Stonecash, 2017, p. 15).

Conspiracy narratives therefore represent a distinct type of mythical discourse which can sometimes be used to “spice up“ one’s political myths but which could actually deprive the myths of force when used without discretion. A good example of this is the behavior of some of the Czech anti-systemic politicians during the COVID-19 pandemic. As in other countries, repeated lockdowns produced a powerful culture of distrust across Czech society, and it was tempting for various political entrepreneurs to tap into it. The most important of them was the controversial MP Lubomír Volný, who in February 2021 tried to unite all smaller nationalist COVID-19-skeptical movements under the Free Block political label. In his anti-COVID-19 fervor, Volný did not shy away from spreading conspiracy narratives (Režňáková & Vachtl, 2021). Thus, on 2 March 2021, he announced on his Facebook page that he had just learned of plans to discredit the ivermectin drug by killing several patients using it. And when two days later a DHL cargo plane circled several times over the city of Brno and then flew away, Volný speculated that the plane had been spraying the British coronavirus mutation “as an artificially created biological weapon.“

Volný’s approach seemed successful at first; in the early months of 2021, his Facebook profile became by far the most followed political profile in the Czech Republic (Tvrdoň & Prchal, 2021). Yet, while this shows the myths narrated by Volný to be highly attractive, their popularity never turned into election support, and in the October 2021 national elections, Volný’s Free Block completely failed, winning only 1.33% of the vote. The reason seems to be precisely its anti-systemic nature, which worked fine enough for articulating popular frustration and generating Facebook likes, but which has failed as a political myth, i.e., as a narrative envisioning a reform of the system instead of its outright rejection. Building one’s political profile only on distrust turned out not to be a good strategy after all.

It is useful to compare Volný’s approach with that of Tomio Okamura, the strongest Czech anti-systemic politician and the leader of the Freedom and Direct Democracy party (of which Volný himself had been a member until March 2019). Okamura is a nationalist-populist famous for his anti-EU stance, and for many years, he has been able to articulate popular frustrations by means of scapegoating anti-migration narratives. We might therefore also expect him to incline toward COVID-19-skeptical and anti-vaxx rhetoric. In fact, however, he has been extremely cautious in this regard, confining himself to criticism of various specific anti-pandemic measures, rejecting compulsory vaccination (as did most Czech political parties), and demanding that antibody tests be allowed as a substitute for vaccination (a reasonable claim supported by many immunologists). In the end, it was only in the dramatic tone with which he raised these claims that Okamura was tapping into popular COVID-19 distrust. In terms of the content of his claims, Okamura was careful not to overdo the distrust. His strategy worked, and in the October election, he won 9.56% of the vote.

The case of Lubomír Volný illustrates the great difference between political myths and conspiracy myths. The latter may deeply resonate with large segments of the public, but their ethos of distrust makes them unsuitable for mobilizing a sufficient number of supporters for common political action. Political mobilization requires at least an elementary acceptance of the political system as such. Yet this is precisely what conspiracy myths undermine. By using them, therefore, politicians run the risk of decreasing the willingness of their supporters to participate in the elections at all.

## Conclusions

I have argued that conspiracy narratives may be conceptualized as a special type of social myth which is different from two other major types, political and fictional myths, though it interacts with both. Conspiracy myths are fictional myths which are taken metonymically, i.e., which are understood by their audience as having a relatively straightforward relation to reality and as providing an authoritative framework for social and political action. Unlike political myths, though, they are anti-systemic at heart, and their chief ethos is that of distrust. Nevertheless, the degree to which they reject the system varies, and it is thus useful to distinguish between weaker and stronger conspiracy myths. While the latter reject the system altogether and are entirely opposed to political myths, the former are capable of co-operating with them, and sometimes, they may even function as their useful complement.

This mythological perspective may help us problematize some stereotypes which are frequently tied to the study of conspiracy theories. First, in common scholarly usage, describing conspiracy narratives as “myths“ implies that these narratives are somehow less rational than those told by mainstream social and political actors. In this way, we are creating an artificial divide between conspiracy narratives and normal political discourse. From my point of view (derived from Gérard Bouchard), all modern political discourse is mythical at heart and is far more “irrational“ and based on unconscious forces and deep mental structures than we commonly admit (something we can easily observe in all election campaigns). It is only once we recognize this fundamental mythical nature of our everyday political thought that we may impartially investigate its similarities with and differences from conspiracy thinking. If, on the other hand, we see conspiracy thinking as a revival of archaic mental patterns altogether different from modern political rationality, we will not be able to explain why these patterns spread so easily. In actuality, both mainstream political myths and conspiracy myths are a mixture of reason and emotion, and what makes the latter different is mainly their anti-systemic nature and their ethos of distrust.

Second, designating conspiracy narratives as “mythical“ frequently implies that they are fantastic and unsubstantiated by facts. Yet, as has often been pointed out, it is frequently difficult to say which conspiracy theory is founded on facts and which is not. The mythological perspective liberates us from such problems by shifting attention from *facts* to *narratives* and to the effects they have on us. As we have seen, mainstream political myths often are based on facts, but this does not make them any less mythical. What matters are the collective emotions and the moral ethos that the narrative in question produces. Whether the narrative is fact-based is often of little importance. After all, some of the most important political myths are purely imaginary ideological constructions: objectively speaking, there are no such entities as “human rights“ or “gender equality,“ yet we still base many of our decisions on them and treat them as something entirely real. In the same manner, a narrated conspiracy is real for those who believe in it despite the fact that it may be purely imaginary. Its reality is performative: it consists not in some external facts to which it refers but in the effects it produces in the audience. Indeed, the very practice of “fact-checking“ is also largely mythical: its chief aim is to discredit rival narratives and validate those we endorse by referring to “facts“ as powerful sources of sacred authority (Cloud, 2019; van der Linden, 2020).

This is not to say that all myths are equal. Still, the difference between them is not to be judged by their facticity but rather by their outcomes. A good social myth is one that mobilizes its listeners toward constructive political action. As we have seen, however, here lies the main weakness of conspiracy myths. Since their basic ethos is that of distrust, in most cases, they lead to resignation and resentment. On the other hand, such an outcome is not inevitable, and there are cases where a conspiracy myth in the end produces good results. Once this happens, the narrative ceases to be a conspiracy myth and becomes a political myth. The best-known example is the US Declaration of Independence, which accused the King of conspiring against the colonists but managed to do so in a manner that allowed the foundation of a new political order (Atkinson & DeWitt, 2019, p. 131). Nevertheless, such a successful transformation is only possible when the conspiracy myth is not too strong and does not reject the system altogether.

## Footnotes

1 Throughout the text, I use the term “myth” whenever I wish to emphasize that the narrative in question possesses these special characteristics that distinguish it from cultural narratives in general. I speak of “narratives” whenever I wish to evoke the basic features of all narratives regardless of whether they function as myths or not (particularly when I want to draw attention to those features that we usually associate with narratives, such as their plot).

2 One of the reviewers of this article wondered how religious myths fit in. My answer is that depending on the myth in question as well as on its audience, they may stretch all across the map. On the vertical axis, they may support the sociocultural system by serving as its legitimizing “charter,“ or they may criticize the system in the manner of millennial myths (gnostic myths being a classic example). On the horizontal axis, they may be regarded as symbolic expressions of divine reality, or may only be read metaphorically as fascinating symbolic illustrations of the human condition. However, radically critical myths and the non-literal readings of myth only emerge in post-Axial religions. Pre-Axial myths have their center of gravity at the upper left pro-systemic metonymic corner of the map, though they also display mild anti-systemic features of the scapegoating kind, telling of various evil powers to be fought.

## References

[CR1] Alexander JC, Smith P, Hall John R, Grindstaff Laura, Lo Ming-Cheng (2010). The strong program: Origins, achievements, and prospects. Handbook of Cultural Sociology.

[CR2] Atkinson MD, DeWitt D, Uscinski Joseph E (2019). The politics of disruption: social choice theory and conspiracy theory politics. Conspiracy Theories and the People Who Believe Them.

[CR3] Aupers S (2012). “Trust no one‘: Modernization, paranoia and conspiracy culture. European Journal of Communication.

[CR4] Barkun M (2003). A culture of conspiracy: Apocalyptic visions in contemporary America.

[CR5] Bennett WL (1975). Political sanctification: The civil religion and american politics. Social Science lnformation.

[CR6] Boltanski, L. (2014). *Mysteries and conspiracies: Detective stories, spy novels and the making of modern societies*. Tr. by Catherine Porter. Cambridge – Malden: Polity Press.

[CR7] Bonetto E, Arciszewski T (2021). The creativity of conspiracy theories. The Journal of Creative Behavior.

[CR8] Bottici C (2007). A philosophy of political myth.

[CR9] Bouchard, G. (2017). *Social myths and collective imaginaries*. Tr. by Howard Scott. Toronto – Buffalo – London: University of Toronto Press.

[CR10] Bouchard G (2013). National myths: Constructed pasts, contested presents.

[CR11] Bratich JZ (2008). Conspiracy panics: Political rationality and popular culture.

[CR12] Brunner J (1991). The narrative construction of reality. Critical Inquiry.

[CR13] Butter, M. (2014). *Plots, designs, and schemes: American conspiracy theories from the puritans to the present*, Berlin – Boston: De Gruyter.

[CR14] Butter, M. (2020). Conspiracy theories in American history. In Michael Butter and Peter Knight (Eds.), *Routledge handbook of conspiracy theories.* London – New York: Routledge, 648–659.

[CR15] Butter, M., & Knight, P. (2020). General introduction. In Michael Butter and Peter Knight (Eds.), *Routledge handbook of conspiracy theories.* London – New York: Routledge, 1–8.

[CR16] Carver, B., Cracium, D., & Hristov, T. (Eds.) (2022). *Plots: Literary form and conspiracy culture*. London – New York: Routledge.

[CR17] Cloud DL, Winkler Carol (2019). Ideology, argument, and the post-truth panic. Networking argument.

[CR18] Dabezies, A. (1992). From primitive myths to literary myths. In Pierre Brunel (Ed.), *Companion to literary myths, heroes and archetypes*. Tr. by Wendy Allatson, Judith Hayward, and Trista Selous. London: Routledge, 960–967.

[CR19] Douglas, M. (1975). Jokes, In *Implicit meanings: Essays in anthropology*. London – New York: Routledge, 90–114.

[CR20] Edelman M (1967). Myth, metaphors and political conformity. Psychiatry.

[CR21] Enders AM, Smallpage Steven M, Uscinski Joseph E (2019). Polls, plots, and party politics: Conspiracy theories in contemporary America. Conspiracy theories and the people who believe them.

[CR22] Fenster, M. (2008). *Conspiracy theories: Secrecy and power in American culture*. Minneapolis – London: University of Minnesota Press.

[CR23] Flood CG (1996). Political myth: A theoretical introduction.

[CR24] Fried RM (1990). Nightmare in red: The McCarthy era in perspective.

[CR25] Frye N (1957). Anatomy of criticism: Four essays.

[CR26] Geertz C, Banton Michael (1966). Religion as a cultural system. Anthropological approaches to the study of religion.

[CR27] Girardet R (1986). Mythes et mythologies politiques.

[CR28] Giry J (2015). Conspiracism: Archaeology and morphology of a political myth. Diogenes.

[CR29] Goertzel T (1994). Belief in conspiracy theories. Political Psychology.

[CR30] Harambam, J. (2020). *Contemporary conspiracy culture: Truth and knowledge in an era of epistemic instability*. London – New York: Routledge.

[CR31] Hoewe J, Brownell KC, Wiemer EC (2020). The role and impact of Fox News. The forum.

[CR32] Hosking G, Schöpflin G (1997). Myths and nationhood.

[CR33] Icke, D. (2001). *Children of The Matrix: How an interdimensional race has controlled the world for thousands of years-and still does*. Wildwood: Bridge of Love.

[CR34] Ivakhiv, A. J. (2018). Occult geographies, or the promises of spectres: Scientific knowledge, political trust, and religious vision at the margins of the modern. In Paul Stenner and Michel Weber (Eds.), *Orpheus’ Glance: Selected Papers on Process Psychology*. Louvain-la-Neuve: Éditions Chromatika, 115–144.

[CR35] Kingdon JW (1984). Agendas, alternatives, and public policies.

[CR36] Knight, P. (2000). *Conspiracy culture: From Kennedy to the X-Files*. London – New York: Routledge.

[CR37] Latour B. (1991). *We have never been modern*. Tr. by C. Porter. Cambridge, MA: Harvard University Press.

[CR38] Lévi-Strauss C (1955). The structural study of myth. The Journal of American Folklore.

[CR39] Lewis T, Kahn R (2005). The reptoid hypothesis: Utopian and dystopian representational motifs in David Icke’s alien conspiracy theory. Utopian Studies.

[CR40] Madisson, M.-L. (2014). The semiotic logic of signification of conspiracy theories. *Semiotica: Journal of the International Association for Semiotic Studies*, 202, 273−300.

[CR41] Malinowski B (1926). Myth in primitive psychology.

[CR42] Marietta M (2008). From my cold, dead hands: Democratic consequences of sacred rhetoric. Journal of Politics.

[CR43] McKenzie-McHarg, A. (2020). Conceptual history and conspiracy theory. In Michael Butter and Peter Knight (Eds.), *Routledge handbook of conspiracy theories.* London – New York: Routledge, 16–27.

[CR44] Melley T (2000). Empire of conspiracy: The culture of paranoia in postwar America.

[CR45] Moore A, Uscinski Joseph E (2019). On the democratic problem of conspiracy politics. Conspiracy theories and the people who believe them.

[CR46] Perry, D. (2017). 10 conspiracy theories that turned Fox News into the Donald Trump channel, *The Oregonian/OregonLive*, 1 November 2017. Available at https://www.oregonlive.com/trending/2017/11/10_conspiracy_theories_that_tu.html.

[CR47] Popper, K. R. (2013) [1945]. *The open society and its enemies*. Princeton, NJ: Princeton University Press.

[CR48] Režňáková, L., & Vachtl, J. (2021). Vraždy kvůli ivermektinu, COVID jako zbraň. Vyvracíme dezinformace Volného, *iDnes.cz*, 9 March 2021. Available at https://www.idnes.cz/zpravy/domaci/dezinformace-lubomir-volny-socialni-site-prispevky-koronavirus-Covid.A210308_102344_domaci_lre

[CR49] Robertson DG, Dyrendal A, Uscinski Joseph E (2019). Conspiracy theories and religion: Superstition, seekership, and salvation. Conspiracy theories and the people who believe them.

[CR50] Roeland, J., Aupers, S., & Houtman, D. (2012). Fantasy, conspiracy and the romantic legacy: Max Weber and the spirit of contemporary popular culture. In Adam Possamai (Ed.), *Handbook of hyper-real religions*. Leiden: Brill, 401–422.

[CR51] Smith AD (1999). Myths and memories of the nation.

[CR52] Sorel, G. (1999) [1908]. *Reflections on violence*. Ed. by Jeremy Jennings. Cambridge: Cambridge University Press.

[CR53] Stonecash, J. M. (2017). Donald Trump and the power of narratives. A paper submitted for the conference *State of the Parties: 2016 and Beyond*, November 9–10, 2017. Available at https://www.uakron.edu/bliss/state-of-the-parties/papers/Stonecash.pdf

[CR54] Tangherlini TR, Shahsavari S, Shahbazi B, Ebrahimzadeh E, Roychowdhury V (2020). An automated pipeline for the discovery of conspiracy and conspiracy theory narrative frameworks: Bridgegate, Pizzagate and storytelling on the web. PLoS One.

[CR55] Thacker, P. D. (2021). The COVID-19 lab leak hypothesis: Did the media fall victim to a misinformation campaign? *BMJ*, 8 July 2021: 374n1656. 10.1136/bmj.n1656 34244293

[CR56] Thalmann K (2019). The stigmatization of conspiracy theory since the 1950s: “A plot to make us look foolish’.

[CR57] Tudor H (1972). Political myth.

[CR58] Tvrdoň, J., & Prchal, L. (2021). Sázka na agresi se Volnému vyplatila. Přitáhl pozornost frustrovaných lidí, na Facebooku přeskočil i Babiše, *Deník N*, 9 March 2021, https://denikn.cz/577914/sazka-na-agresi-se-volnemu-vyplatila-pritahl-pozornost-frustrovanych-lidi-na-facebooku-preskocil-i-babise/.

[CR59] Uscinski JE, Uscinski Joseph E (2019). What is a conspiracy theory?. Conspiracy theories and the people who believe them.

[CR60] Uscinski, J. E. (2020). *Conspiracy theories: A primer*. Lanham: Rowman, & Littlefield.

[CR61] Uscinski JE, Parent JM (2014). American Conspiracy Theories.

[CR62] van der Linden S, Panagopoulos C, Roozenbeek J (2020). You are fake news: Political bias in perceptions of fake news. Media, Culture, & Society.

[CR63] van Prooijen J-W, Krouwel APM, Pollet TV (2015). Political extremism predicts belief in conspiracy theories. Social Psychological and Personality Science.

